# A descriptive analysis of food pantries in twelve American states: hours of operation, faith-based affiliation, and location

**DOI:** 10.1186/s12889-022-12847-0

**Published:** 2022-03-17

**Authors:** Natalie D. Riediger, Lindsey Dahl, Rajeshwari A. Biradar, Adriana N. Mudryj, Mahmoud Torabi

**Affiliations:** 1 Department of Food and Human Nutritional Sciences, Faculty of Agricultural and Food Sciences, University of Manitoba, 209 Human Ecology Building, Winnipeg, MB Canada; 2 Department of Community Health Sciences, Rady Faculty of Health Sciences, University of Manitoba, Winnipeg, MB Canada; 3 Manitoba Centre for Health Policy, Department of Community Health Sciences, Max Rady College of Medicine, Rady Faculty of Health Sciences, University of Manitoba, Winnipeg, MB Canada; 4 Tata Institute of Social Sciences, Mumbai, India; 5 Department of Epidemiology and Biostatistics, KLE Academy of Higher Education & Research (K.A.H.E.R) Nehru Nagar, Belagavi, Karnataka India

**Keywords:** Food pantry, Food bank, Food security, Rural, Religion, Faith-based

## Abstract

**Background:**

Our objectives were to describe both the development, and content, of a charitable food dataset that includes geographic information for food pantries in 12 American states.

**Methods:**

Food pantries were identified from the foodpantries.org website for 12 states, which were linked to state-, county-, and census-level demographic information. The publicly available 2015 Food Access Research Atlas and the 2010 US Census of Population and Housing were used to obtain demographic information of each study state. We conducted a descriptive analysis and chi-square tests were used to test for differences in patterns of food pantries according to various factors.

**Results:**

We identified 3777 food pantries in 12 US states, providing an estimated 4.84 food pantries per 100,000 people, but ranged from 2.60 to 7.76 within individual states. The majority of counties (61.2%) had at least one food pantry. In contrast, only 15.7% of all census tracts in the study states had at least one food pantry. A higher proportion of urban census tracts had food pantries compared to rural tracts. We identified 2388 (63.2%) as being faith-based food pantries. More than a third (34.4%) of food pantries did not have information on their days of operation available. Among the food pantries displaying days of operation, 78.1% were open at least once per week. Only 13.6% of food pantries were open ≤1 day per month.

**Conclusions:**

The dataset developed in this study may be linked to food access and food environment data to further examine associations between food pantries and other aspects of the consumer food system (e.g. food deserts) and population health from a systems perspective. Additional linkage with the U.S. Religion Census Data may be useful to examine associations between church communities and the spatial distribution of food pantries.

## Introduction

Food insecurity refers to the inability to access sufficient, safe, and nutritious food to meet the dietary needs and food preferences for an active and healthy life [1]. The prevalence of household food insecurity in 2019 in the United States was estimated at 10.5% [2], which has only been exacerbated during the COVID-19 pandemic [3]. Given the significant number of food insecure households and food insecurity’s strong associations with poor health outcomes [4–6], particularly diabetes [7, 8], and increased health care costs [9], food insecurity is a major public health concern. While food insecurity is inextricably linked to low income, food-based interventions at the municipal-level, such as food pantries have been thrust to the forefront in an attempt to alleviate the problem [10]. Food banks refer to charitable food assistance organizations that rely upon food and monetary donations in order to either distribute food to smaller charities that serve food insecure populations, or to provide a direct grocery service to clients, sometimes called food pantries or food shelves [11].

The inception of charitable food organizations in the 1960s was intended to serve as emergency food aid in response to short-term food insecurity. By the 1990’s, emergency food aid had grown to such an extent that in Detroit, Michigan there were more food banks, pantries, and soup kitchens (*N* = 100) than supermarkets and large grocery stores (*N* = 96) [12]. Despite the rise in charitable food, there is a lack of evidence supporting their effectiveness in addressing the main issue of food insecurity. At the individual-level, the charitable food system has been shown to contribute to stigma and shame among patrons [13–15], offer poor nutritional value [11, 16], provide insufficient and inconsistent food supply [11–17], consist of limited food choice and variety [16], and exacerbate pre-existing chronic health conditions [11, 18, 19]. Furthermore, “pantries spring up wherever someone is moved to create them” [20] (p221). In this way, the geographical distribution of food pantries may not follow any systematic pattern or necessarily reflect need. Many food pantries operate out of churches and volunteers are often motivated to volunteer because of their religious commitments. Given these circumstances and undercurrents, faith is an important and dynamic element of the charitable food system. However, faith-based affiliations within the current charitable food system is unknown and likely context-specific.

While the experiences of clients of the charitable food system have been explored qualitatively [13, 21, 22], and there is considerable individual-level data to evaluate food security programs, there has been little, if any, ecological data to describe the charitable food *system.* The charitable food system is one component, or sub-system, of the larger consumer food system, as well as part of the broader social and economic system. The scope of the charitable food system is related to overall food security, food security programs (i.e., food stamps), both smaller and larger grocery stores, and religious communities (i.e., churches), as some examples. Taken together, all these sub-systems also influence health outcomes (e.g., diabetes). An ecological analysis applying systems theory [23] as a conceptual framework to examine the consumer food system could provide important policy-relevant evidence regarding the charitable food system, as well as publicly-funded food security programs, food security, and health. Ecological studies are especially useful when the implications for intervention are at the population- or systems-level.

From a systems theory perspective [24, 25], we understand that if charitable food makes up an increasing component of the consumer food system, other aspects of the food or economic systems counterbalance for this increase. For example, the reliance on the charitable food sector has reduced the pressure on governments to improving income security through social programs [26, 27], and may further reduce participation in other public food programs. Similarly, applying a systems perspective, reductions in churches or declining participation in faith-based communities [28, 29] may diminish charitable food assistance. The increasing involvement of the corporate food sector through donations (supported by governmental tax programs) may further aggravate food system inequality by contributing to the dissolution of smaller grocers and the preponderance of “food deserts” or areas devoid of fresh and whole foods in disadvantaged neighborhoods [30]. Smaller businesses may be unable to provide food at a comparable price to either larger grocery stores or the charitable sector, which is free. The interconnectedness of the food system is further displayed through the food systems impacts from the COVID-19 pandemic [3]. In this way, systems theory may be useful for exploring structural issues and inequities within consumer food systems, including charitable food systems as a sub-system.

In order to empirically examine the relationships amongst the consumer food sub-systems for future research, we have created a *Charitable Food Dataset* (CFD), which lists and documents characteristics of charitable food organizations in select states in the U.S. The objectives of this paper are to (1) describe the developed dataset and (2) describe the charitable food system according to days of operation, faith-based affiliation, and rural/urban location. This methods paper describes a dataset that can be linked to other publicly available datasets to further explore relationships within the food system.

## Methods

### Data sources

The CFD was constructed primarily from the publicly available charitable food organization directory at foodpantries.org [31]. The website is not affiliated with any governmental agency or non-profit organization, and manually collects information on food pantries, soup kitchens and non-profit organizations (collectively referred to as ‘food pantries’ hereinafter) in the 50 US states and the District of Columbia. Food pantries were identified in 12 study states (Alabama, Georgia, Illinois, Indiana, Kansas, Kentucky, Louisiana, Michigan, Mississippi, Ohio, Tennessee and West Virginia) purposefully selected to achieve variation in the sample based on state prevalence of food security listed in the 2018 Food Environment Atlas, the inclusion of both rural and urban areas, as well as the number of food pantries in each state. The CFD contains the name, address, and hours/days of operation of each food pantry in the directory, which were entered into Microsoft Excel. The data were cleaned and any entries that shared the same name and/or address were considered duplicates and removed. The CFD data were collected between February 2017 and July 2018, and are stored in an open source repository [32].

Census tract numbers for each food pantry in the study states were obtained using the United States Census Bureau’s (USCB) Geocoder address look-up tool [33] and the one-line address associated with each food pantry. If census tracts were not found using the Geocoder, the food pantry was geographically located using Google Maps [34] and cross-referenced with the corresponding USCB Census Tract Reference Map [35]. The reference maps are county-based and display the census tract numbers for each delineated tract area within that county for the 2010 Census, which was the latest census completed in the US.

The publicly available 2015 Food Access Research Atlas [36] and the 2010 US Census of Population and Housing [37] were also used to obtain demographic information of each study state. The Food Access Research Atlas is maintained by the United States Department of Agriculture (USDA), and contains food access indicators at the census-tract level.

### Variables

The Food Access Research Atlas includes the 2010 populations of each census tract based on the estimates from the 2010 US Census. These estimates were used to estimate the populations for each study state. The Atlas also includes an indicator variable for urban or rural census tracts based on whether the geographic centroid of the census tract is in an area with more than 2500 people as determined by the 2010 block-level population data and aerially allocated to ½ kilometre square grids [38]. This indicator variable was used to calculate the populations and the number of census tracts stratified by urban and rural census areas for each state. The number of counties per state was determined by converting the census tract numbers provided in the Atlas into county Federal Information Processing Standards (FIPS) and removing any duplicates. Finally, the 2010 Census was used to obtain the square mile of land area in each state as a measure of its geographic size.

A variable indicating whether a food pantry was affiliated with a Judeo-Christian organization was created by reviewing each food pantry name in the CFD for Judeo-Christian terms, for example, “Saint” or “church” (Table 1). These terms were subjectively selected based on their likelihood of being recognized as Judeo-Christian terms by someone who was using the foodpantries.org directory. We refer to these food pantries as faith-based for the remainder of the paper. Lastly, the days of operation of food pantries contained in the CFD varied widely ranging from 4 days per year to 5 days per week. Therefore, a variable was created by collapsing the days of operation into 5 categories: (1) ≤1 day per month; (2) 2–3 days per month; (3) once per week; (4) 2–3 days per week; (5) ≥4 days per week.

**Table 1:** Faith-based key words or terms used to identify faith-based food pantries

Key words/Terms	Meaning^a^
Saint/St.	Christian person recognized has holy
God	Supreme Being of power, wisdom, and goodness who is worshipped as creator and ruler of the universe
Grace	Virtue coming from God
Baptist	One who admits a person to the Christian community
Methodist	Member of one of the denominations deriving from the Wesleyan revival in the Church of England
Protestant	Member of any of several Christian church denominations other than Catholic or Eastern church
Adventist	Member of any denomination whose system of belief is in the second coming of Christ
Ministry/Ministries	A group ordained to perform pastoral functions in a Christian church
Helping Hands	N/A
Madonna	The Virgin Mary
Jesus	The Jewish religious teacher whose life, death, and resurrection as reported by the Evangelists are the basis of the Christian message of salvation
Church	Building for public and especially Christian worship
Catholic	Of, relating to, or forming the church universe
Evangelical/Evangelist	Of, relating to, or being in agreement with the Christian gospel
Temple	House of worship for Jewish congregations
Salvation Army	Deliverance from the power and effects of sin
Fishes and Loaves	A Christian parable referring to Jesus feeding a multitude of people

^a^The meanings of key words/terms are adapted from definitions provided in the Merriam-Webster dictionary

### Analysis

Descriptive statistics were generated for the study states, including population counts, number of counties, number of census tracts, proportion of urban and rural census tracts, and land area. Population counts for each state are from 2018, with the most recent estimates calculated from the 2010 Census. The urban and rural populations were determined by using the 2010 Census, which were provided at the census-tract level in the Food Access Research Atlas.

Descriptive analyses, using the state demographic information, were conducted for the food pantries in the CFD. First, the number of food pantries in each state was counted and the proportions that were in urban and rural census tracts were calculated. Second, the number of food pantries per 100,000 state population stratified by urban and rural tracts, as well as the number of food pantries per 1000 square miles of state land area, were determined. Third, the numbers of counties, census tracts, urban tracts, and rural tracts that had at least one food pantry were calculated, and the proportions within urban compared to rural census tracts were tested using chi-square tests. Fourth, the number and proportion of food pantries per state that were faith-based were calculated, and then stratified by urban and rural areas. The proportion of faith-based pantries in urban areas was compared with the proportion in rural areas using chi-square tests. Lastly, the frequencies within each category of days of operation were calculated. Chi-square tests were used to explore differences in the distributions of faith-based and non-faith-based food pantries, and of urban and rural food pantries, in the days of operation categories. The level of significance was set at 0.05 for all statistical tests, which were carried out using the Statistical Package for Social Science (SPSS) version 23.

## Results

The selected states represent a total of 1112 counties and 19,167 census tracts (Table 2). The average state population was 6,507,685 (SD = 3,642,347) and ranged from 1,852,994 to 12,830,632 people. The majority of census tracts were considered to be located in urban areas (69.1%), which represented approximately two thirds of the total population in the study states. The average state land area was 47,796 square miles (SD = 14,403) with a minimum of 24,038 and a maximum of 81,759 square miles.

**Table 2:** Study state population, number of counties, census tracts, and land area

State	Food insecurity (2013–15)^a^	2010 Population^b^ (n)			Counties^a^ (n)	Census Tracts^b^ (n (%))			Land Area (sq. miles)^c^
		State	Urban	Rural		Total	Urban	Rural	
Alabama	17.6	4,779,736	2,569,178	2,210,558	67	1179	644 (54.6)	535 (45.4)	50,645
Georgia	14.9	9,687,653	6,815,195	2,872,458	159	1965	1319 (67.1)	646 (32.9)	57,513
Illinois	13.8	12,830,632	10,886,519	1,944,113	102	3121	2625 (84.1)	496 (15.9)	55,519
Indiana	11.1	6,483,802	4,437,690	2,046,112	92	1508	1043 (69.2)	465 (30.8)	35,826
Kansas	14.6	2,853,118	2,006,947	846,171	105	770	508 (66.0)	262 (34.0)	81,759
Kentucky	17.6	4,339,367	2,253,898	2,085,469	120	1115	557 (50.0)	558 (50.0)	39,486
Louisiana	18.4	4,533,372	3,103,403	1,429,969	64	1143	816 (71.4)	327 (28.6)	43,204
Michigan	14.9	9,883,640	7,104,264	2,779,376	83	2774	2007 (72.4)	767 (27.6)	56,539
Mississippi	15.2	2,967,297	1,356,005	1,611,292	82	662	329 (50.0)	333 (50.3)	46,923
Ohio	16.1	11,536,504	8,627,924	2,908,580	88	2949	2283 (77.4)	666 (22.6)	40,861
Tennessee	15.1	6,346,105	3,924,941	2,421,164	95	1497	896 (60.0)	601 (40.1)	41,235
West Virginia	11.3	1,852,994	779,059	1,073,935	55	484	217 (44.8)	267 (55.2)	24,038
Total Mean SD	N/A	78,094,220 6,507,682 3,642,505	53,865,023 4,488,752 3,176,214	24,229,197 2,019,100 680,171	1112 93 28	19,167 1597 910	13,244 (69.1) 1104 795	5923 (30.9) 494 166	573,548 47,796 14,403

Sources: ^a^ 2018 Food Environment Atlas;^b^ 2015 USDA Food Access Research Atlas [29]; ^c^ 2010 Census of Population and Housing [30]

We identified 3923 food pantries using the foodpantries.org directory, with 47 of the entries requiring the USCB reference maps to locate their census tract numbers. We removed 146 duplicate food pantry entries, resulting in 3777 individual food pantries in the CFD with three quarters of them located in urban census tracts (Table 3). The number of food pantries per 100,000 people in the overall sample was 4.84, ranging from 2.60 to 7.76 within the individual states. There were 5.31 food pantries per 100,000 in urban census tracts, and 3.79 in rural census tracts, and there were 6.59 food pantries per 1000 square miles of land area. The majority of counties (61.2%) had at least one food pantry. In contrast, only 15.7% of all census tracts in the study states had at least one food pantry (Table 4). Significantly more urban census tracts had a food pantry compared to rural census tracts (16.8% vs. 13.3%; *p* < .00001). We identified 2388 (63.2%) as being faith-based food pantries, with a significantly higher proportion of urban food pantries being faith-based compared to rural food pantries (65.1% vs. 57.4%, *p* < .0001; Table 5).

**Table 3:** Number of food pantries, and the proportion of food pantries per 100,000 people in the population in each study state, and in each study state stratified by urban and rural census tracts

	Food Pantries, n (%)			Food Pantries Per 100,000 Population^a^			Food Pantries per 1000 sq. miles
State	Total	Urban	Rural	Total	Urban	Rural	
Alabama	358	223 (62.3)	135 (37.7)	7.49	8.68	6.11	7.07
Georgia	326	240 (73.6)	86 (26.4)	3.37	3.52	3.00	5.67
Illinois	473	391 (82.7)	82 (17.3)	3.69	3.59	4.22	8.52
Indiana	503	370 (73.6)	133 (26.4)	7.76	8.34	6.50	14.04
Kansas	92	76 (82.6)	16 (17.4)	3.22	3.79	1.89	1.13
Kentucky	120	77 (64.2)	43 (35.8)	2.77	3.42	2.06	3.04
Louisiana	174	142 (81.6)	32 (18.4)	3.84	4.58	2.24	4.03
Michigan	734	587 (80.0)	147 (20.0)	7.43	8.26	5.29	12.98
Mississippi	156	108 (69.2)	48 (30.8)	5.26	7.96	2.98	3.32
Ohio	552	461 (83.5)	91(16.5)	4.78	5.34	3.13	13.51
Tennessee	165	112 (67.9)	53 (32.1)	2.60	2.85	2.19	4.00
West Virginia	124	73 (58.9)	51 (41.1)	6.69	9.37	4.75	5.16
Total	3777	2860 (75.7)	917 (24.3)	4.84	5.31	3.79	6.59

^a^ population counts from the 2010 Census of Population and Housing as per the 2015 USDA Food Access Research Atlas [29]

**Table 4:** Number and proportion of counties and census tracts with at least one food pantry in each study state, and in each study state stratified by urban and rural census tracts

State	Counties with a Food Pantry, n (%)	Census Tracts with a Food Pantry, n (%)			
		Total	Urban	Rural	*p*-value
Alabama	59 (88.1)	273 (23.2)	165 (25.6)	108 (20.2)	0.028
Georgia	85 (53.5)	269 (13.7)	193 (14.6)	76 (11.8)	0.082
Illinois	59 (57.8)	389 (12.5)	317 (12.1)	72 (14.5)	0.131
Indiana	85 (92.4)	406 (26.9)	293 (28.1)	113 (24.3)	0.125
Kansas	32 (30.5)	76 (9.9)	62 (12.2)	14 (5.3)	0.003
Kentucky	51 (42.5)	95 (8.5)	57 (10.2)	38 (6.8)	0.041
Louisiana	31 (48.4)	149 (13.0)	119 (14.6)	30 (9.2)	0.014
Michigan	69 (83.1)	563 (20.3)	435 (21.7)	128 (16.7)	0.004
Mississippi	52 (63.4)	128 (19.3)	83 (25.2)	45 (13.5)	< 0.001
Ohio	60 (68.2)	433 (14.7)	358 (15.7)	75 (11.3)	0.005
Tennessee	58 (61.1)	141 (9.4)	91 (10.2)	50 (8.3)	0.233
West Virginia	39 (70.9)	88 (18.2)	50 (23.0)	38 (14.2)	0.013
Total	680 (61.2)	3010 (15.7)	2223 (16.8)	787 (13.3)	< 0.001

**Table 5:** Number and proportion of faith-based food pantries in each study state, and in each study state stratified by urban and rural census tracts

Food Pantries, n (%)				
State	Faith-based	Urban*Faith-based	Rural*Faith-based	*p*-value
Alabama	256 (71.5)	173 (77.6)	83 (61.5)	0.001
Georgia	205 (62.9)	165 (68.8)	40 (46.5)	< 0.001
Illinois	286 (60.5)	246 (62.9)	40 (48.8)	0.017
Indiana	321 (63.8)	238 (64.3)	83 (62.4)	0.693
Kansas	50 (54.5)	43 (56.6)	7 (43.8)	0.349
Kentucky	74 (61.7)	52 (67.5)	22 (51.2)	0.077
Louisiana	103 (59.2)	83 (58.5)	20 (62.5)	0.674
Michigan	488 (66.5)	390 (66.4)	98 (66.7)	0.958
Mississippi	111 (71.2)	74 (68.5)	37 (77.1)	0.276
Ohio	340 (61.6)	292 (63.3)	48 (52.7)	0.058
Tennessee	91 (55.2)	66 (58.9)	25 (47.2)	0.156
West Virginia	63 (50.8)	40 (54.8)	23 (45.1)	0.288
Total	2388 (63.2)	1862 (65.1)	526 (57.4)	< 0.001

More than a third (34.4%) of food pantries did not have information on their days of operation available. The proportion of faith-based versus non-faith based food pantries that did not provide this information was not significantly different (35.4% vs. 32.7%; *p* = 0.093), as was the case for urban and rural food pantries (33.9% vs. 36.2%; *p* = 0.202). Among the food pantries displaying days of operation, 52.9% were open at least 2 days per week, while 78.1% were open at least once per week (Table 6). Only 13.6% of food pantries were open ≤1 day per month. Significant relationships existed between the days open categories and whether the food pantry was faith-based (*p* < .00001), or was located in an urban or rural area (*p* = 0.043). A higher proportion of faith-based and rural food pantries fell into the less frequently open categories, and had a lower proportion in the more frequently open categories as compared to non-faith-based food pantries and urban food pantries, respectively.

**Table 6:** Distribution of food pantries according to days of operation, stratified by faith-based and non-faith based food pantries, and urban and rural census tracts, separately

	Food Pantries, n (%)				
Number of days per week	Total (*n* = 2479)	Faith-based (*n* = 1549)	Non-faith based (*n* = 930)	Urban (*n* = 1894)	Rural (*n* = 585)
≤1 day per month	336 (13.6%)	270 (17.4%)	66 (7.1%)	241 (12.7%)	95 (16.2%)
2–3 days per month	208 (8.4%)	160 (10.3%)	48 (5.2%)	158 (8.3%)	50 (8.6%)
Once per week	624 (25.2%)	451 (29.1%)	173 (18.6%)	465 (24.6%)	159 (27.2%)
2–3 days per week	649 (26.2%)	364 (23.5%)	285 (30.7%)	518 (27.4%)	131 (22.4%)
≥4 days per week	662 (26.7%)	304 (19.6%)	358 (38.5%)	512 (27.0%)	150 (25.6%)
*p*-value		<.001		0.043	

## Discussion

In this study, we described the processes involved in developing the CFD, a dataset containing information on food pantries in 12 US states. Descriptive findings indicate approximately three quarters of food pantries are located in urban areas, and almost two thirds were considered to have a faith affiliation, which were also more common in urban versus rural areas. Among pantries with hours of operation posted, 78.1% were open at least 1 day per week, and non-faith-based and urban food pantries were more likely to be open more often. This dataset can be linked via FIPS to a number of publicly available datasets, such as the USDA Food Access Research Atlas, the USDA Food Environment Atlas, and the U.S. Religion Census Data. Through linkage of this CFD with other datasets, a number of research questions can be examined.

Food insecurity affected 10.5% of households in the United States in 2020, and is more common among households with children, and Black or non-Hispanic householders [39]. Given the prevalence of food insecurity, efforts to mitigate food insecurity have the capacity to greatly improve population health at multiple levels – national, state, county, household, and individual. The role of food assistance programs has increased as a result of the decline of non-food social programs. The largest publicly-funded food assistance program in the US is SNAP, which provided ‘food stamp’ benefits to more than 44 million people in 2016 [40]. However, many eligible people do not participate in the program, and among those who do, approximately half of them continue to report being food insecure [41]. Charitable food assistance programs or food banks, which were initially established to provide emergency food supplies, are now considered to supplement the governmental programs in their effort to address food insecurity. In fact, 26.5% of food insecure households and 4.8% of all US households used a food pantry in 2016, representing a 40 and 68% increase from 2001, respectively [42]. For these reasons, it has become increasingly important to consider the effects of growing charitable food programs on food security and health [43].

Charitable food organizations, and other community-level initiatives, have the potential to improve individual health through emergency food provision [10]. However, most health research, media attention, and governmental policy action is disproportionately focused on individual health and exposures, which limits our ability to understand structural drivers of inequality [43–45]. The USDA’s Food Access Research Atlas and the Food Environment Atlas provide data on food access and environmental indicators at the census tract- and county-levels. By assigning census tract numbers to each food pantry, the CFD is able to link with both atlases, providing an opportunity to explore the structural drivers of health inequality as it relates to food pantries, ‘food deserts’, federal food programs, food insecurity, and health. The CFD is also able to link with US Religion Census data [46], which contains data on congregations, members, adherents, and attendees, or the population purported to sustain the charitable food sector.

The CFD consists of more food pantries located in urban census tracts compared to rural tracts, reflecting the higher proportion of urban census tracts in the US. The proportion of urban census tracts that had at least one food pantry was 26% higher compared to rural census tracts, and the number of food pantries per urban population was higher than the number per rural population by approximately 40%. This is inconsistent with previously reported data using county-level information from the *Map the Meal Gap* project and the Hunger in America 2014 survey, which showed that the number of charitable food locations per 1000 people was highest in counties that were considered completely rural according to urban-rural continuum codes [47]. However, the difference in defining urban and rural areas (i.e., county versus census tract) makes it difficult to compare the findings from the two studies. Given that food insecurity is more prevalent in more populated metropolitan areas compared to nonmetropolitan areas, this may indicate that the food pantries in our dataset are located in areas of greatest need [3]. However, further research is needed to provide estimates of food insecurity at the census-tract level in order to determine if, in fact, the food pantries are concentrated in the areas that would benefit most from their service.

Charitable food organizations rely heavily on food and monetary donations, and volunteers for their operation. In this way, faith-based or religious organizations, with their ability to engage their communities and which often work for social justice and against inequality, are set up well to provide such services [48]. In addition, volunteerism in food banks and pantries is often motivated by faith and has an important role in building community [20]. This may explain the higher proportion of food pantries identified as faith-based in the CFD. The relationship between religion and population health has been extensively explored, and through its ability to provide social capital to communities, especially the most vulnerable communities, illustrate religions’ importance as a social determinant of health [49, 50]. However, the variability in the hours of operation of food pantries reflects the volunteer nature of food banking, and is a legitimate concern among clients given that many rely on prolonged use of food pantries [4, 51]. This may illustrate the limits of volunteerism in addressing food insecurity, which may be exacerbated as participation in faith-based communities is declining [29].

The large size and diversity of the CFD is a strength, which provides a foundation for future research exploring the relationship between charitable food and social- and health- related outcomes from a systems perspective. However, there are several limitations of this dataset, and the present study, that must be considered. First, the completeness of the dataset is uncertain. The foodpantries.org directory only contains information regarding updates, corrections, or new food pantries that they receive manually through an online submission form. Some food pantries may not be in the directory, while others may still be included despite closure. We documented 146 directory entries that were considered duplicates because they shared either the same name or address with another entry, which illustrates the limitation in the maintenance of the directory. Furthermore, Feeding America advertises 200 food banks and 60,000 food programs as part of its network; however, foodpantries.org documented only 15,494 food pantries in total in 2018, with 3777 included in the present study. While it is unclear how Feeding America defines a ‘food program’ or whether the 60,000 food programs are a cumulative or a point prevalence, there is clearly a large discrepancy. However, our documented totals for food pantries in Detroit, Michigan are very similar to previous research conducted 4 to 5 years earlier; in addition to foodpantries.org, authors also utilized Local Harvest and several local sources to identify food pantries [12].

Second, faith-based affiliations were subjectively determined using a collection of common Judeo-Christian terms, which may have led to some misclassification. This approach identified 63.2% of food pantries as Christian faith-based. In her 1998 book, Poppendieck states that “more than 70 percent of the pantries and kitchens affiliated with the Second Harvest Network are sponsored by churches or other religious organizations” and that this is likely an underestimate of “the prevalence of religious orientation” [20] (p188–89).

Third, while many food pantries are open at least once a week, the quantity of food available per family, their form (pre-packaged food boxes or grocery store style), and the quality of the food provided is unknown. Research suggests that the quality of food available at food pantries does not meet recommendations put in place by health professionals [52]. Furthermore, we are missing data on days of operation for nearly a third of food pantries.

Fourth, food pantries that are only open a few times a year (i.e., one to four times) are also included in the foodpantries.org directory. These food pantries likely operate only during specific holidays (i.e., Christmas and Thanksgiving); while they can address immediate hunger, they will have limited impact on individual or population-level food insecurity.

Fifth, the proportion of food pantries per population used the most recent population estimates from the US Census that could be stratified by urban and rural census areas, which were estimated 8 years prior to the date that the number of food pantries was determined. The populations increase slightly each year, therefore, the proportions are likely over-estimated. Lastly, the inclusion of only 12 states may limit the generalizability of the data to the United States as a whole, though it is also unlikely that the 12 states selected are completely unique to the country.

To validate the completeness of the dataset, extensive ground truthing exercises and/or comparison to other existing local datasets collected through other means could be completed. This may mitigate some of the limitations previously described. This dataset could be updated through identical methods, and corresponding validation procedures. Ideally, all countries with charitable food systems, particularly those receiving public funds, should be keeping public records or datasets of food pantries to track the distribution of charitable food. Ensuring accurate and complete data is critical to informing policy related to food security.

## Conclusions

In conclusion, food pantries in these 12 states are mostly set in urban areas, and affiliated with Judeo-Christian organizations. Their operation hours vary considerably; however, many are open at least once a week. The dataset developed in this study may be linked to food access and food environment data to further explore associations between concentration of food pantries and other aspects of the consumer food system and prevalence of health outcomes, such as diabetes, from a systems perspective. Additional linkage with the U.S. Religion Census Data may be useful to examine associations between church communities and the spatial distribution of food pantries. The number of food pantries has increased since their inception in the 1960s, which may be attributed to ongoing deficiencies in publicly-funded food security and social programs. However, the extent to which the existence of food pantries is dependent on faith-based communities and associated volunteers is concerning given the precarity of operations and declines in church participation [29]. We must understand the implications of the charitable food system to the larger food and economic system, prior to continuing to grow the charitable sector, either directly with funding, or indirectly through reduced spending to social programs.

## Data Availability

All data sources used in this study are publicly available. Riediger, Natalie, 2022, “Charitable Food Descriptors located in 12 States in America”, 10.34990/FK2/GHL06P, University of Manitoba, V1, UNF:6:B8jxycLhATZEObfR8BW2mQ== [fileUNF]. United States Department of Agriculture, Economic Research Service. Food Access Research Atlas. 2017. https://www.ers.usda.gov/data-products/food-access-research-atlas/. Accessed November 10, 2021. United States Census Bureau. United States Summary: 2010. Population and Housing Unit Counts. 2012. https://www.census.gov/prod/cen2010/cph-2-1.pdf . Accessed November 11, 2021.

## Abbreviations

CFD: Charitable Food Dataset
SNAP: Supplemental Nutrition Access Program
USCB: United States Census Bureau
USDA: United States Department of Agriculture
WIC: Women, Infants, and Children program
